# Exploration of sodium homeostasis and pharmacokinetics in bile duct-ligated rats treated by anti-cirrhosis herbal formula plus spironolactone

**DOI:** 10.3389/fphar.2023.1092657

**Published:** 2023-01-18

**Authors:** Tun-Pin Hsueh, Tung-Hu Tsai

**Affiliations:** ^1^ Department of Chinese Medicine, Kaohsiung Chang Gung Memorial Hospital and Chang Gung University College of Medicine, Kaohsiung, Taiwan; ^2^ School of Chinese Medicine, Chang Gung University, Taoyuan, Taiwan; ^3^ Institute of Traditional Medicine, School of Medicine, National Yang Ming Chiao Tung University, Taipei, Taiwan; ^4^ Graduate Institute of Acupuncture Science, China Medical University, Taichung, Taiwan; ^5^ School of Pharmacy, Kaohsiung Medical University, Kaohsiung, Taiwan

**Keywords:** Yin-Chen-Hao-Tang, spironolactone, cirrhosis, ascites, canrenone, reninangiotensin–aldosterone system

## Abstract

Renal sodium retention is an essential indicator that is used for the prognosis of cirrhosis with ascites that requires diuretic treatment to restore sodium homeostasis. The diuretic effects of Yin-Chen-Hao-Tang (YCHT) alone or in combination with diuretics for sodium retention in patients with cirrhosis have not been investigated. This study aimed to investigate the diuretic effects and sodium retention caused by YCHT with spironolactone, from both the pharmacokinetic and pharmacodynamic perspective, in bile duct-ligated rats. The HPLC method was validated and utilized for the pharmacokinetic analysis of rat urine. Urine samples were collected and analyzed every 4 hours for 32 h after oral administration of YCHT at 1 or 3 g/kg daily for 5 days in bile duct-ligated rats. A dose of 20 mg/kg spironolactone was also administered to pretreat the YCHT 1 g/kg or the 3 g/kg group on the 5th day to explore the interaction of the two treatments. Urine sodium, potassium, weight, volume, and spironolactone and canrenone levels were measured to investigate fluid homeostasis after the coadministration. The linearity, precision, and accuracy of the HPLC method were suitable for subsequent urinary pharmacokinetic analyses. The pharmacokinetic parameters in the 1 g/kg YCHT with spironolactone group revealed that the elimination half-life of the spironolactone metabolite, canrenone, was prolonged. In addition, the cumulative excretion amount, the area under the rate curve (AURC), and the maximum rate of excretion (Rmax) were significantly decreased when the spironolactone group was pretreated with 3 g/kg YCHT. Urinary sodium excretion elicited by spironolactone was suppressed by pretreatment with 1 or 3 g/kg YCHT. The 32-hour urine output was not altered by the administration of YCHT alone, but it was significantly decreased by 64.9% after the coadministration of YCHT with spironolactone. The interaction of spironolactone and YCHT was found to decrease urine sodium–potassium and water excretion, and this change was attributed to the decreased level of spironolactone metabolites and possibly the regulation of the renin–angiotensin–aldosterone system by obstructed cirrhosis. The dose adjustment of YCHT or diuresis monitoring should be noted when co-administering YCHT and spironolactone to treat hepatic diseases clinically.

## 1 Introduction

Ascites, as part of decompensation syndrome, is a survival factor in end-stage liver disease (Wiesner et al., 2003). These commonly observed symptoms occur during chronic cirrhosis, and end-stage cirrhosis was responsible for a 50% mortality rate at 5 years in patients who did not undergo a liver transplant (Planas et al., 2006). Portal hypertension is a prerequisite for the development of ascites, leading to clinical manifestations of tympanites or edema. Although ascites is the first and most common symptom of cirrhosis and indicates a poor prognosis, it is different from that of patients with mild sodium retention, normal renal function, and a good response to diuretics (Kamath et al., 2007). Marked sodium retention was found to be a prognostic factor in cirrhosis with ascites early in 1981 (Arroyo et al., 1981), and sodium retention has been identified as the most reliable indicator of poor prognosis, accompanied by low arterial pressure and low urine sodium levels (Sersté et al., 2012). The association between sodium excretion and reduced survival has been confirmed, and this parameter has been used as a guide for prognosis in cirrhosis with ascites.

Renal sodium retention in the collecting ducts or distal tubules is primarily promoted by secondary hyperaldosteronism due to excessive activation of the renin–angiotensin–aldosterone system (RAAS) (Wilkinson et al., 1979). Activation of RAAS leads to the leakage of excessive blood volume directly from the liver surface and sodium retention, which is driven by angiotensin II and relative hypoalbuminemia. Sodium and water retention processes usually occur prior to ascites development. Therapeutic options, including dietary salt restriction or diuretic therapy, can help to achieve the cornerstone of ascites treatment for negative sodium homeostasis. However, dietary salt restriction alone has limited efficacy for patients because a less palatable diet could increase non-compliance rates and worsen malnutrition. Primary diuretic treatment with aldosterone antagonist spironolactone or canrenone is administered stepwise to help maintain sodium restriction (Moore et al., 2003). Spironolactone is considered a better natriuretic than loop diuretics such as furosemide, with a recommended initial dose of 100–200 mg once daily (Perez-Ayuso et al., 1983; Angeli et al., 1994). Although diuretic spironolactone can achieve negative sodium retention, dilutional hyponatremia or hyperkalemia may be a concern because of the non-osmotic secretion of antidiuretic hormones (Angeli et al., 2006).

Yin-Chen-Hao-Tang (a decoction of *Artemisia capillaris*, YCHT) is composed of the following three botanical drugs, namely, *Artemisia capillaris* Thunb., *Gardenia jasminoides* J.Ellis, and *Rheum officinale* Baill. with a ratio of 4:3:3. This formula was developed two thousand years ago in the “Treatise on Febrile Diseases” for damp-heat jaundice due to expelling heat and dampness in the traditional Chinese medicine theory. It was also used in Japan called Inchinko-to for severe acute icteric hepatitis (Ohwada et al., 2009). *Artemisia capillaris* Thunb (*A. capillaris*), an essential herbal medicine in YCHT, has been shown to have many therapeutic effects, including antihepatitis, antifibrosis, and anticancer effects, as well as antidiabetic and antiviral properties, which contribute to its bioactive compounds (Hsueh et al., 2021). Adding the other two herbal medicines, *Gardenia jasminoides* J. Ellis (*G. jasminoides*) and *Rheum officinale* Baill *(Rheum)*, potentially enhances the pharmacological effects of the bioactive compounds in *A. capillaris* (Hsueh and Tsai, 2018). The three herbal medicines that compose YCHT were found to exert a protective effect against chlorpromazine-induced cholestatic liver injury (Yang et al., 2015) and suppress oxidative stress, leading to anti-fibrogenesis (Zhang et al., 2016). YCHT has been used clinically for liver diseases ranging from hepatitis to severe liver cancer due to its anti-inflammatory, antiviral, and antifibrotic effects, ability to repair hepatic injury, and reversal of steatosis (Li et al., 2017). Despite the application of YCHT in various liver diseases, few studies have investigated sodium and water homeostasis in liver cirrhosis.

Decompensated cirrhosis develops due to liver inflammation, hepatitis, fibrosis, cirrhosis, or cancer. Sodium and water retention processes were already present before ascites developed. Primary diuretic treatment with spironolactone is considered a better natriuretic agent to maintain sodium restriction. YCHT has been used to treat hepatitis, cirrhosis, and liver cancer, owing to its hepatoprotective properties. However, the therapeutic effects of this herbal formula on sodium retention remain unknown. In addition, patients with decompensated cirrhosis could be on concurrent treatment with YCHT and the diuretic drug spironolactone with unknown consequences. The benefits of diminishing sodium retention from combined YCHT effects on liver disease or spironolactone in preventing ascites could be contradictory. However, the net effects of YCHT and spironolactone on sodium and water homeostasis have not yet been investigated. This study aimed to investigate sodium and water homeostasis effects of YCHT alone and in combination with spironolactone from both pharmacokinetic and pharmacodynamic perspectives to explore the possible pharmaceutical interactions between these medicines in cirrhotic rats to achieve better simultaneous use of herbal medicines and diuretics in future preclinical applications.

## 2 Materials and methods

### 2.1 Chemicals and reagents

Pharmaceutical herbal formula powder Yin-Chen-Hao-Tang (YCHT) was purchased from Koda Pharmaceutical Co., Ltd., (Taoyuan, Taiwan). *Artemisia capillaris* Thunb*.*, *Gardenia jasminoides* J.Ellis, and *Rheum officinale* Baill were made from the crude drug at a ratio of 18:6:6 with an extract of 6.0 g (crude drug and extract ratio 30:6 = 5:1), according to the pharmaceutical company’s instructions. Diuretic spironolactone was purchased from Sigma‒Aldrich Research Biochemicals, Inc., (St. Louis, MO, United States). The pharmaceutical herbal formula powder YCHT was quantified using a UHPLC–MS/MS analysis system coupled to an electrospray ionization (ESI) source equipped with a triple quadrupole mass spectrometer (LCMS-8030 system; Shimadzu, Kyoto, Japan) to guarantee consistent quality of the herbal formula powder, which contained .207 mg/g of scoparone, 7.241 mg/g of geniposide, and .093 mg/g of rhein (Hsueh et al., 2016). The metabolite of spironolactone canrenone (purity ≥97%) and the internal standard n-propylbenzene for urinary pharmacokinetic analysis were both obtained from Sigma‒Aldrich Inc., (St. Louis, MO, United States). Other chemicals for analysis, including formic acid (98%–100%) and ethanol, were of analytical grade and purchased from Merck (Darmstadt, Germany).

### 2.2 HPLC method validation

Urinary samples for pharmacokinetic analysis were detected using an LC-20 high-performance liquid chromatography (HPLC) system (Shimadzu, Kyoto, Japan), consisting of a system controller (CBM-20A), pumps (LC-20AD XR), degasser (DGU-20A3), autosampler (SIL-20 AC XR), and column oven (CTO-20A) coupled with a UV detector (SPD-M20A). A reverse-phase C18 column (Purospher STAR, 100 mm × 2.1 mm, 2 μm, Merck, Darmstadt, Germany) was equipped, and the separation of the analytes was performed using .1% formic acid aqueous solution–MeOH (43:57, v/v) at a flow rate of .2 mL/min, as well as a total run time of 15 min. The UV wavelength was 254 nm for detection. Method validation of bioanalytical assays for urinary pharmacokinetic experiments was based on the bioanalysis guidelines of the US FDA (Meesters and Voswinkel, 2018).

### 2.3 Animals and experimental design

The adult male Sprague Dawley (SD) rats used in this study were obtained from the Laboratory Animal Center at National Yang Ming Chiao Tung University (Taipei, Taiwan), and the animal experiments were approved by the Institutional Animal Experimentation Committee of National Yang Ming Chiao Tung University (IACUC approval number 1050503). Rats (6, 7 weeks old, 250 ± 50 g) were provided free access to food and water and housed in a pathogen-free environment. All animal experiments followed the guidelines and procedures of laboratory animal care at National Yang Ming Chiao Tung University.

To test the solitary diuretic effects of YCHT in bile duct-ligated (BDL) rats, the rats were anesthetized with 50 mL/kg pentobarbital (10 ml/kg body weight). The bile duct was ligated from both sides, and the midpoint of the bile duct was cut, followed by wound suturing. BDL-operated rats were maintained on a standard diet and tap water *ad libitum* and were randomly assigned to experimental YCHT groups that were orally administered 1 g/kg or 3 g/kg of YCHT daily for 5 days or a control group administered the same volume of distilled water.

For the investigation of the impact of YCHT on the diuretic drug spironolactone, the BDL rats were assigned to four groups, including the YCHT groups that were orally administered 1 g/kg or 3 g/kg of YCHT daily for 5 days, which corresponds to a daily intake of 9.7 g or 29.2 g, respectively, for a 60-kg human. The spironolactone and vehicle groups received the same volume of distilled water at the same time. These four groups, except for the vehicle group, were administered 20 mg/kg spironolactone via gavage on the 5th day.

### 2.4 Urine samples for pharmacodynamics

Rats were housed in metabolic cages after spironolactone administration. Urine samples of each group were also collected at 0–4, 4–8, 8–12, 12–16, 16–20, 20–24, and 24–32 h. To explore how pretreatment with YCHT affected diuretic drugs, urine weight, volume, and sodium and potassium concentrations were monitored at each collection interval. The sodium concentration divided by the potassium concentration corresponded to the effects of spironolactone and showed 75% sensitivity and 92% specificity (El Basel et al., 2015). Urinary sodium and potassium levels were determined using an automated urinalysis analyzer in a clinical laboratory.

### 2.5 Sample preparation for urinary pharmacokinetic analysis

Urine samples from each interval were prepared using a liquid‒liquid extraction procedure, followed by high-performance liquid chromatography-ultraviolet (HPLC-UV) analysis. The urine sample at each interval was spiked with an internal standard, added to ethyl acetate, vortexed for 5 min, and centrifuged for 10 min at 13,000 rpm at 4°C. After repeating the procedure twice to achieve effective phase separation, the supernatant was collected and transferred to a rotary evaporator for an hour. Finally, the residues were remixed in 50% methanol and injected into HPLC equipment for analysis.

### 2.6 Urinary pharmacokinetic analysis

The spironolactone metabolite, canrenone, in urine was measured to investigate the pharmacokinetic interaction of YCHT with spironolactone. Canrenone excreted in urine was calculated by urine volume plus each collection period to plot the cumulative amount of canrenone (Cum). The estimated percentage of the cumulative amount of canrenone excreted versus the administration dose was calculated and presented as Cum %. Urinary excretion rates were determined by dividing the entire dosing interval by 4-h intervals and referred to as the maximum observed excretion rate (Rmax). Therefore, the associated time of Rmax (Tmax) was determined from the excretion rate data. The area under the urinary excretion rate curve represents the individual urinary excretion rate over time and was estimated from zero to the last sampling (AURC_0– 32_) and infinite time (AURC _0–∞_). The elimination half-life (t_1/2_) is the product of ln2 divided by the elimination rate constant (Ke). The estimated urinary pharmacokinetic parameters from the profiles were calculated using WinNonlin version 1.1 (Scientific Consulting Inc., Apex, NC, United States) with a non-compartmental model.

### 2.7 Statistical analysis

Data acquired in the experiment are presented as the mean ± standard deviation (S.D.). One-way analysis of variance (ANOVA) was used to compare more than two groups to analyze the differences in means among the groups. The unpaired two-tailed t-test was used for comparisons between the two groups. Differences were defined as statistically significant when p values were lower than .05.

## 3 Results

### 3.1 Urinary pharmacokinetic interaction of Yin-Chen-Hao-Tang and spironolactone

**FIGURE 1:**
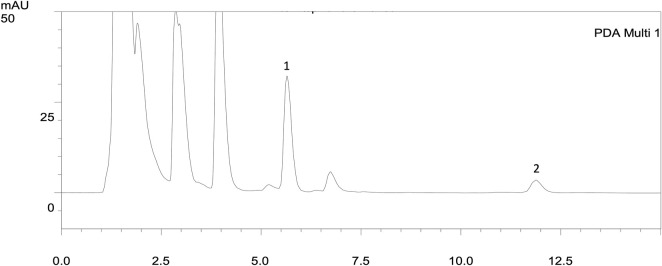
HPLC chromatograms of urine extracts after administration of spironolactone in 16 h (20 mg/kg, p.o.). The parent drug spironolactone was not detected in the urine extract. 1: n-Propylbenzene (IS, RT: 5.61 min); 2: canrenone (RT: 11.11 min).

The developed HPLC method achieved good linearity (y = .047x + .0081, *R* ^2^ > .999) of canrenone from .5 μg/mL (the lowest limit of quantitation, LLOQ) to 50 μg/mL, and thus it was suitable to be applied for the pharmacokinetic study of rat urine. The parent drug spironolactone was not detectable in rat urine as previously reported (Overdiek and Merkus, 1987), but its metabolite canrenone was detected (Figure 1). The developed analytical method for canrenone concentrations in rat urine guaranteed reproducibility with precision and accuracy within ±15% of the nominal values. The precision and accuracy for the intraday assay ranged from .05%–10.76% and −7.28%–4.07%, respectively, and for the interday assay ranged from 1.48%–11.29% and −5.21%–2.05%, respectively (Table 1). The extraction recovery of canrenone in rat urine was assessed in three quality control samples, which represented low, medium, and high concentrations (.25, .5, and 50 μg/mL), and the results revealed consistent and precise values (Table 2). The stability of canrenone in rat urine was consistently independent under long-term, short-term, and auto-sampler conditions (Table 3). The results showed good reproducibility for pharmacokinetic quantification using this analytical method in rat urine.

**TABLE 1:** Intraday and interday precision and accuracy of canrenone in urine samples.

Nominal concentration (μg/mL)	Observed concentration (μg/mL)	Precision (%)	Accuracy (%)
**Intraday**			
0.5	.52 ± .06	10.76	4.07
1	.93 ± .04	4.46	−7.28
5	4.98 ± .19	3.80	−.31
10	10.09 ± .22	2.17	.85
50	49.99 ± .02	.05	−.01
**Interday**			
0.5	.47 ± .05	11.29	−5.21
1	.96 ± .07	7.79	−4.09
5	4.85 ± .17	3.59	−2.93
10	10.21 ± .20	1.92	2.05
50	49.67 ± .74	1.48	−.66

Data are expressed as the means ± S.D.s (*n* = 5).  Precision: RSD (%) = [standard deviation/C_obs_] × 100%; accuracy: bias (%) = [(C_obs_ - C_nom_)/C_nom_] × 100%.

**TABLE 2:** Extraction recovery of canrenone.

Nominal concentration (μg/mL)	Set 1	Set 2	ME (%)^1^
Canrenone			
.25	6629.7 ± 502.6	6532.3 ± 617.5	98.5 ± 4.8
5	97849.3 ± 4711.6	93958.0 ± 7049.3	96.0 ± 4.2
50	1325256.0 ± 63757.5	1306088.0 ± 45146.0	98.6 ± 1.2

Data are expressed as the mean ± S.D. (*n* = 3). Set 2: the mean peak area of the analytes spiked before extraction. Set 1: the mean peak area of analytes spiked post-extraction^1^.

ME: matrix effect (%) calculated as (Set 2/Set 1) × 100%.

**TABLE 3:** Stability of canrenone in rat urine.

Nominal concentration (μg/mL)	Long term	Short term	Autosampler
Canrenone			
0.5	85.72 ± .18	94.37 ± 2.38	95.86 ± 3.86
5	85.65 ± .35	95.94 ± 2.29	95.64 ± 3.87
50	86.61 ± .42	93.20 ± 2.19	95.98 ± 2.61

Data are expressed as the mean ± S.D. (*n* = 3). There was no significant difference by ANOVA.

**FIGURE 2:**
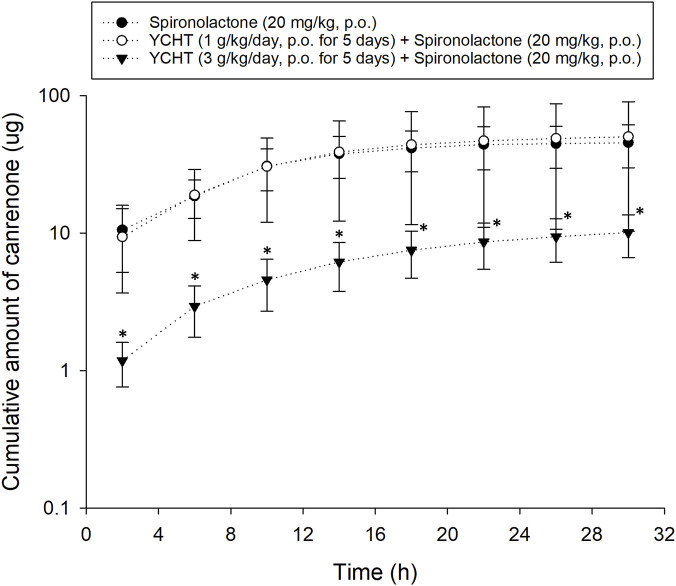
Cumulative amount of canrenone excreted in bile duct-ligated rat urine. The cumulative urinary amount of canrenone versus the time midpoint illustrated the amount of canrenone excreted after using spironolactone, pretreatment with 1 g/kg Yin-Chen-Hao-Tang, and pretreatment with 3 g/kg Yin-Chen-Hao-Tang groups. *p-value < .05 compared to the vehicle group.

Pharmacokinetic experiments were performed to assess the interactions between the herbal formula and diuretics. The mean cumulative amount of the metabolite of spironolactone canrenone was 45.56 µg and 50.28 µg in the spironolactone group and 1 g/kg YCHT with spironolactone group, respectively. However, the mean cumulative amount of canrenone significantly decreased in the 3 g/kg YCHT with spironolactone group during the entire urine sample collection period (Figure 2). Canrenone was found to significantly decrease to 10.11, accounting for only .05% of the dosing for overall 32 h (Table 4). The maximum rate of excretion (Rmax) was also noted to be significantly diminished as .**52** μg/h in the 3 g/kg YCHT with spironolactone group, while it reached 3.42 μg/h and 3.33 μg/h in the other two groups. However, the time to maximum rate (Tmax) was consistent among the three groups. The area under the rate curve (AURC), which consisted of the Rmax versus time plot, was also significantly lower in the 3 g/kg YCHT with spironolactone group. The half-life (t1/2) increased to 6.5 h in the 1 g/kg YCHT with spironolactone group compared to 4.89 h in the spironolactone group. Otherwise, the t1/2 was 11.86 h in the 3 g/kg YCHT with spironolactone group.

**TABLE 4:** Urinary pharmacokinetic parameters of canrenone after oral administration of spironolactone in bile duct-ligated rats.

Parameter	Spironolactone (BDL)	1 g/kg YCHT + spironolactone (BDL)	3 g/kg YCHT + spironolactone (BDL)
Cum (mL µug/mL)	45.56 ± 15.66	50.28 ± 39.95	10.11 ± 3.47**
Cum% (% kg)	.23 ± .08	.25 ± .20	.05 ± .02**
R_max_ (µg/hr)	3.42 ± 1.26	3.33 ± 2.10	.52 ± .21**
T_max_ (hr)	8.0 ± 3.35	6.7 ± 3.01	9.3 ± 5.89
R_avg_ (µg/hr)	1.42 ± 1.0	1.57 ± .98	.22 ± .10*
AURC_0-32_ (mL µug/mL)	42.5 ± 14.4	47.2 ± 37.8	9.5 ± 3.3**
AURC _0-∞_ (mL µug/mL)	43.9 ± 15.0	51.0 ± 43.0	12.4 ± 3.9**
K_e_ (hr^−1^)	.15 ± .03	.11 ± .02*	.06 ± .02
t_1/2_ (hr)	4.89 ± .82	6.50 ± 1.13*	11.86 ± 2.73**

Data are expressed as the mean ± S.D. (n = 6). Data were significant by t-test. *p-value < .05; **p-value < .01. AURC, _0–32_ & AURC, _0-∞_, area under the rate of excretion versus midpoint of time interval curve for time 0–32 h and up to infinity, respectively; Cum, cumulative amount of canrenone excreted within 32 h; Cum%, percent of cumulative amount excreted versus administration dose; Ke, elimination rate constant; R_avg_, average rate of excretion; R_max,_ maximum rate of excretion; T_max_, time to maximum rate of excretion; t_1/2_, elimination half-life; YCHT, Chinese herbal formula Yin-Chen-Hao-Tang.

### 3.2 Pharmacodynamics of Yin-Chen-Hao-Tang in urine

**FIGURE 3:**
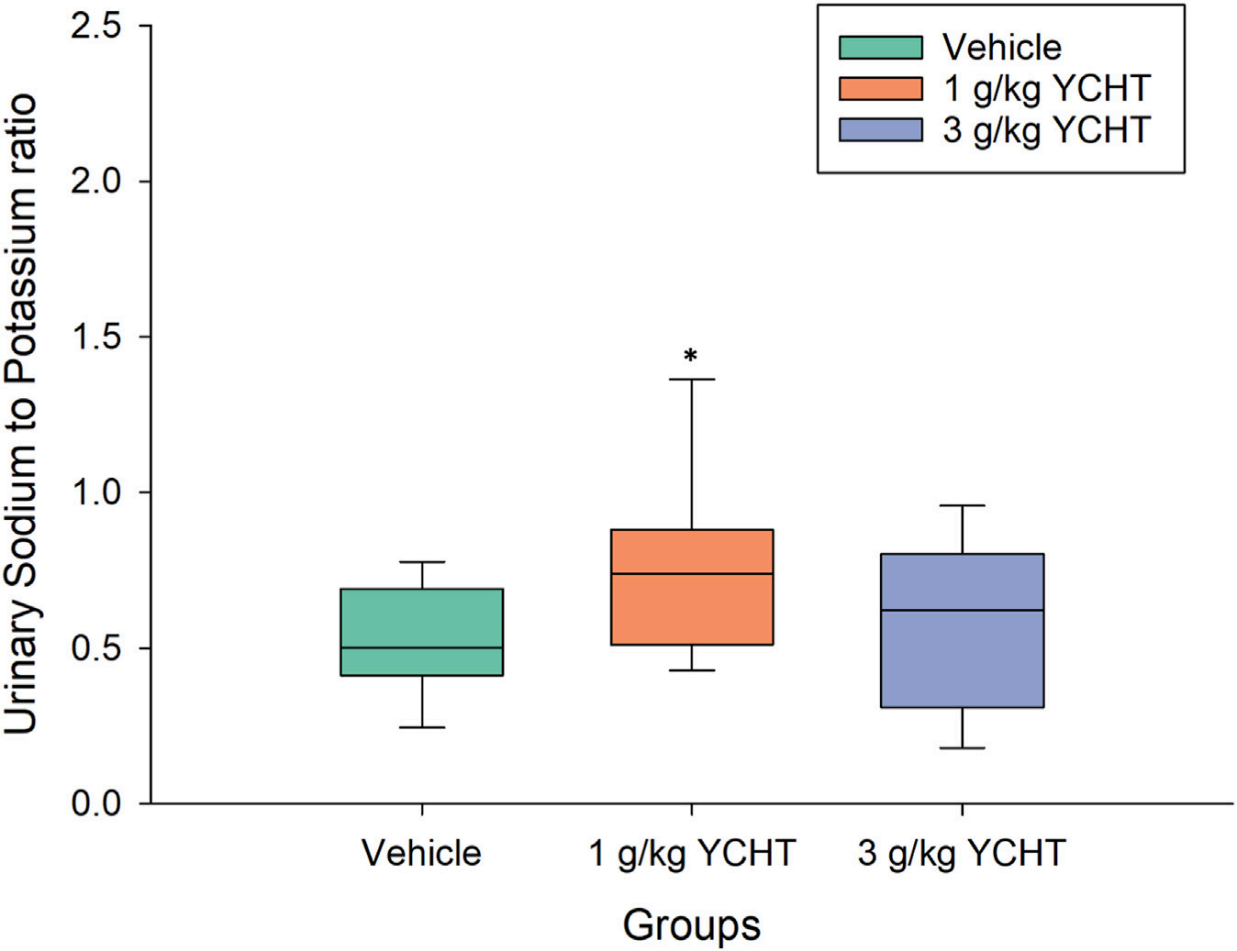
Urine sodium-to-potassium ratio of the vehicle and YCHT treated rats over 32 h. Data were significantly different by t-test. *p < .05; significance compared to the vehicle group; YCHT, Yin-Chen-Hao-Tang.

The total urine amount over 32 h averaged 20.9 mL, 16.8 mL, and 27.3 mL in the vehicle, 1 g/kg, and 3 g/kg YCHT groups, respectively. There were no significant differences in total urine weight or volume among the three groups. However, the urinary sodium–potassium ratio over 32 h was .11–.93 mmol/L in the vehicles, while that of 1 g/kg YCHT ranged from .27 to 1.95 mmol/L, and .09 to 1.19 in 3 g/kg YCHT groups. A significant increase in the sodium–potassium ratio was detected in the 1 g/kg YCHT group compared to that in the vehicle group within 32 h (Figure 3).

### 3.3 Pharmacodynamic interaction of Yin-Chen-Hao-Tang and spironolactone in urine

**FIGURE 4:**
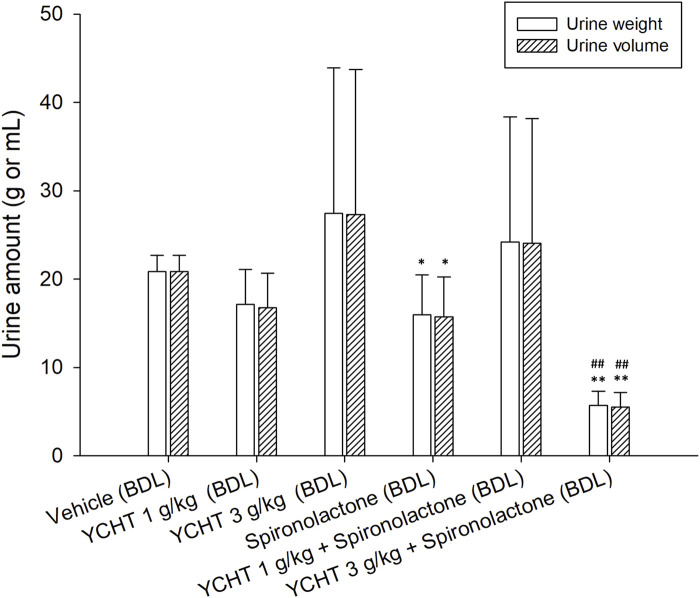
Total urine weight and volume after oral administration of spironolactone in each group. Data were significantly different by t-test. *p < .05 and **p < .01; significance compared to the vehicle group; ##*p*-value < .01; significance compared to the spironolactone group; YCHT, Yin-Chen-Hao-Tang.

After the administration of the diuretic drug spironolactone to bile duct-ligated rats, the urine volume significantly decreased to 15.74 mL (*p* = .03). The urine volume over 32 h remained insignificantly changed to 24.03 mL after treatment with 1 g/kg YCHT and spironolactone. However, the urine volume significantly decreased by 73.5% to 5.53 mL in the 3 g/kg YCHT with spironolactone group compared to that in the vehicle group (Figure 4).

**FIGURE 5:**
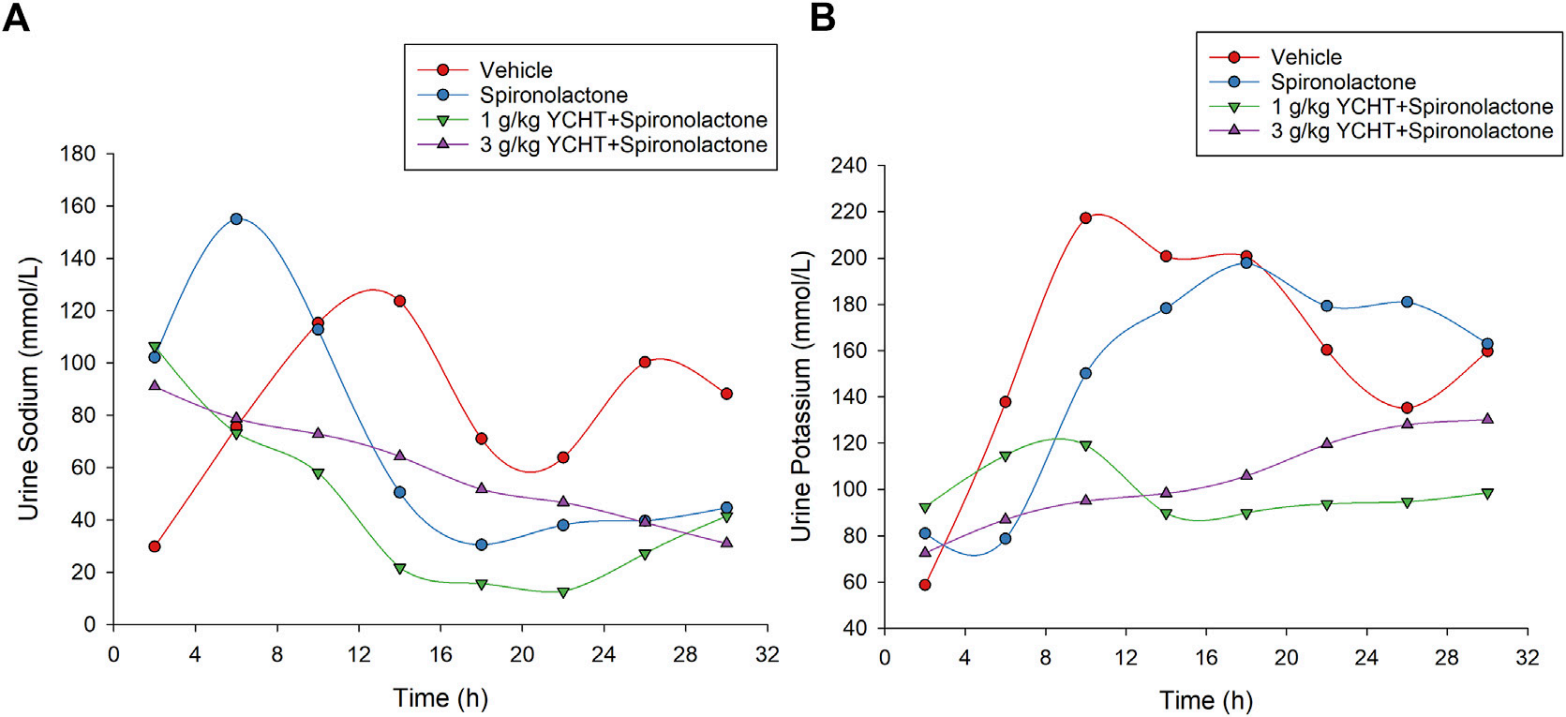
Urine sodium and potassium in the vehicle, spironolactone, 1 g/kg YCHT with spironolactone, and 3 g/kg YCHT with spironolactone groups during 32 h. **(A)** The urine sodium was higher for the first 4 hours in the spironolactone, 1 g/kg YCHT with spironolactone, and 3 g/kg YCHT with spironolactone groups. The urine sodium in the spironolactone group reached the highest level during 4–8 h, while it was decreased in the 1 g/kg YCHT and 3 g/kg YCHT with spironolactone groups. **(B)** The urine potassium in the spironolactone group was lowest during 4–8 h and then elevated as compared to the vehicle groups to 32 h. The urine potassium in the 1 g/kg YCHT and 3 g/kg YCHT with spironolactone groups ranged from 72.5 to 130.2 mmol/L over 32 h.

The urine sodium levels of the spironolactone, 1 g/kg YCHT with spironolactone, and 3 g/kg YCHT with spironolactone groups were significantly elevated for the first 4 hours and were 102.2, 106.5, and 91.0 mmol/L, respectively. The urine sodium level in vehicles was relatively low for the first 4 h and then ranged from approximately 63.8 mmol/L to 123.7 mmol/L for the next 24 h. However, urine sodium level varied from 4–8 h to 155 mmol/L on an average in the spironolactone group but 73.2 mmol/L and 78.7 mmol/L in the 1 g/kg YCHT and 3 g/kg YCHT with spironolactone groups, respectively (Figure 5A). The urine sodium level then all decreased with the lapse of time and reached a mean of 44.6 mmol/L, 41.5 mmol/L, and 31 mmol/L in spironolactone, 1 g/kg YCHT, and 3 g/kg YCHT with spironolactone groups while that of 88.2 mmol/L in the vehicles at 32 h (Figure 5A).

The urine potassium levels otherwise initially ranged from 58.7 to 92.5 mmol/L among these four groups for the first 4 h. Then, the urine potassium level of the vehicles elevated to the highest level, but that of the spironolactone groups decreased to the lowest level from 4 to 8 h among the four groups. The urine potassium levels of the vehicle and spironolactone groups were close to 160 mmol/L at 32 h. There was no significant difference in urine potassium levels between spironolactone and the vehicles at each interval over 32 h. In the others, the urine potassium ranged from 89.8 to 119.2 mmol/L and 87.0–130.2 mmol/L over 4–32 h in the 1 g/kg YCHT and 3 g/kg YCHT with spironolactone groups. Urine potassium levels were significantly lower in the 1 g/kg YCHT with spironolactone group than those in the vehicle group at 32 h (Figure 5B).

**FIGURE 6:**
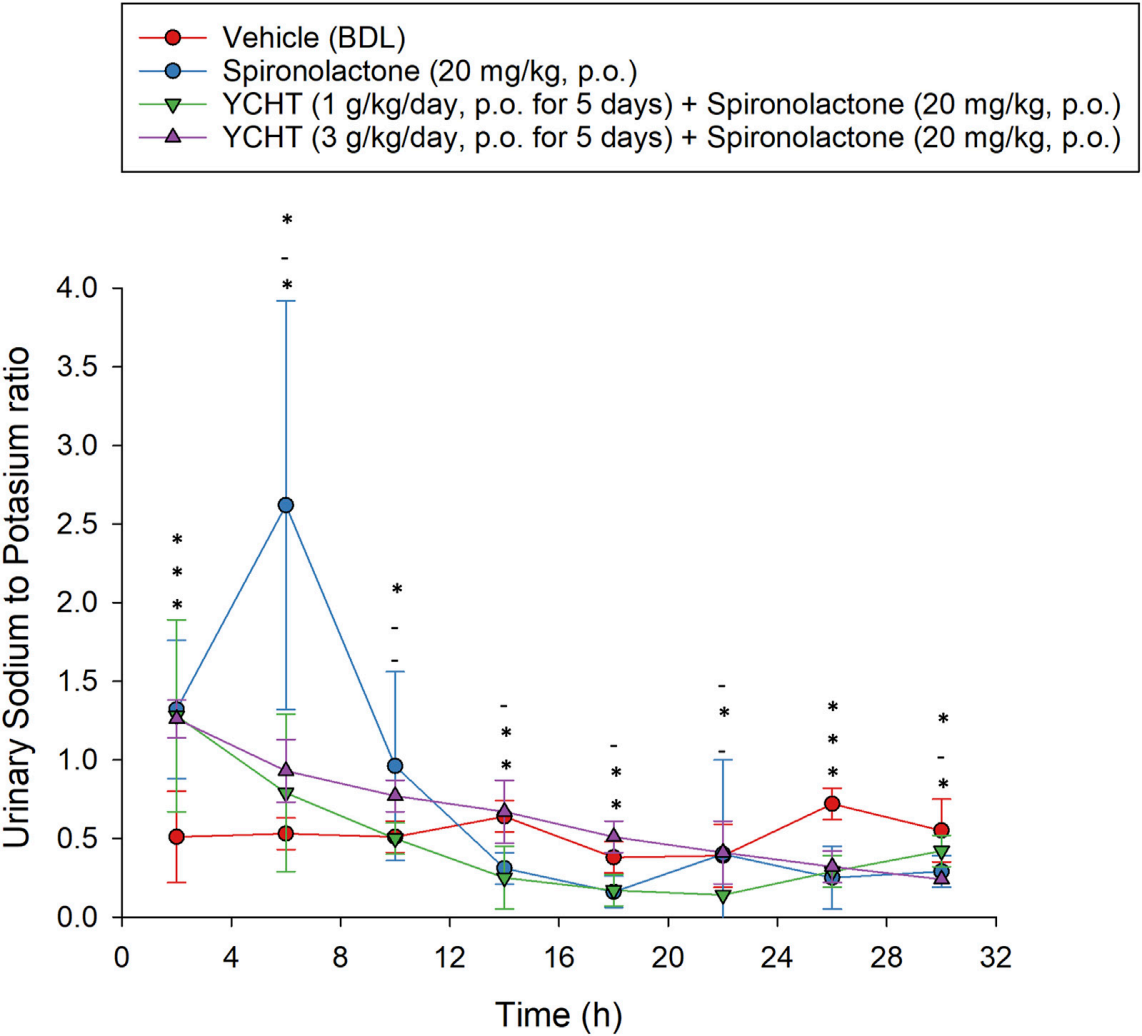
Urine sodium-to-potassium ratio after the single oral administration of spironolactone in each group. Symbols from the bottom to the top represent the spironolactone, 1 g/kg YCHT with spironolactone, and 3 g/kg YCHT with spironolactone groups. *Significance (*p*-value < .05) compared with the vehicle group; - indicates no significance compared to the vehicle group. Data were significantly different by t-test. YCHT, Yin-Chen-Hao-Tang.

For the combination of urine sodium and potassium among the four groups, the sodium-to-potassium ratio was similar to the urine sodium trend flow. The urinary sodium-to-potassium ratio was significantly elevated after a single administration of spironolactone for the first 4 h and reached its highest level of 2.62 in the spironolactone group (Figure 3). The urinary sodium-to-potassium ratio of the 1 g/kg YCHT with spironolactone group was significantly decreased compared to that of the vehicle group after 12 h until 28 h. In contrast, the urinary sodium-to-potassium ratio of the 3 g/kg YCHT with spironolactone group was close to that of the vehicle after 12 h–24 h. The addition of spironolactone seems to alter urinary sodium-to-potassium ratio levels differently between 1 and 3 g/kg YCHT (Figure 6).

## 4 Discussion

This study is the first to investigate the effects of the anti-cirrhosis formula YCHT and its interaction with the diuretic drug spironolactone on sodium homeostasis in bile duct-ligated rats. The developed HPLC method guaranteed good reproducibility for urinary pharmacokinetic quantification by method validation in rat urine. The urinary pharmacokinetics revealed consistent parameters between spironolactone and 1 g/kg YCHT with the spironolactone group, except for a prolonged elimination half-life. The prolonged elimination half-life without increasing the cumulative metabolite canrenone may imply a competition of delayed metabolism of spironolactone by 1 g/kg YCHT, but it is still under the metabolic capacity that leads to the steady canrenone output. In contrast, the cumulative excretion of canrenone significantly decreased when spironolactone was applied after pretreatment with 3 g/kg YCHT, as well as the Rmax and AURC. Furthermore, the elimination half-life increased, revealing interference in the metabolism of spironolactone by YCHT. In addition, despite the alteration of the pharmacokinetic parameters in 3 g/kg YCHT with spironolactone, the excretion Tmax remained consistent among the three groups, indicating that the metabolite fadeout did not occur in the excretion phase.

The 32-hour urine output was not altered by the administration of YCHT alone but by the coadministration of YCHT with spironolactone. Although the urine sodium-to-potassium ratio was elevated in the 1 g/kg YCHT group, the consistent total urine output with the vehicles showed no diuretic effect of YCHT and is unrelated to urinary sodium excretion. In contrast, the diuretic effect of spironolactone was based on acting in sodium–potassium exchange channels to increase sodium and water excretion. The urine sodium of the spironolactone group reached the highest level from 4 to 8 h, which corresponded to an Rmax of 3.42 μg/h in pharmacokinetic parameters. A single administration of spironolactone without continuous excretion of urine sodium might sequentially lead to a decrease in urinary sodium excretion and 32-h urine output in our study. Pretreatment with 1 g/kg YCHT, however, delayed the elimination half-life of canrenone and suppressed urinary sodium excretion elicited by spironolactone, which obtained a normal urine output. On the other hand, pretreatment with 3 g/kg YCHT also suppressed urinary sodium excretion, which could be attributed to the decreasing excretion of cumulative canrenone from the pharmacokinetic results and led to a significant drop in the total urine output.

In addition, the interaction between spironolactone and YCHT leading to changes in the urine output and sodium-to-potassium ratio in bile duct-ligated rats may not be confined only to changes in the metabolite. A previous interaction study showed that treatment with high-dose YCHT and spironolactone led to increasing canrenone excretion in normal SD rats (Hsueh and Tsai, 2020) but decreased urine canrenone excretion was noticed in our study in bile duct-ligated rats, while the combination of high-dose YCHT and spironolactone promoted a decrease in the urine output. In addition to the hepatic injury decreasing the metabolism of administered spironolactone and YCHT, the difference in the combined YCHT with spironolactone treatment in bile duct-ligated rats was the induction of systemic arterial vasodilation and activation of arterial baroreceptors. Hemodynamics are alterations in liver fibrogenesis that cause arterial underfilling with resultant neurohumoral activation, leading to increased RAAS activity, which is a key to maintaining body fluid regulation (Bansal et al., 2009). Recent research on spironolactone has discovered that it participates in the process of antifibrosis formation as an aldosterone receptor antagonist (Zhang et al., 2014). Spironolactone intervention was observed to reverse the decreasing angiotensin-converting enzyme-2 (ACE2) expression induced by obstructive jaundice in rats. The overexpression of renin, angiotensin II, and aldosterone decreased, while angiotensin-(1–7) (Ang-(1–7)) increased after spironolactone administration, which implied that Ang-(1–7) stimulated Mas, a G protein-coupled receptor, leading to anti-inflammatory and antifibrotic effects (Kong et al., 2019). In addition, YCHT treatment was also found to increase the protein expression of ACE2 while maintaining angiotensin-converting enzyme (ACE), angiotensin II, and angiotensin II type 1 receptor (AT1R) levels in the BDL model, leading to the promotion of the ACE2-Ang-(1-7)-MAS pathway for antifibrosis (Wu et al., 2015). Both spironolactone and YCHT were found to enhance the ACE2 expression, leading to Mas activation for anti-inflammatory and antifibrotic effects, but only spironolactone decreased aldosterone and angiotensin II, while YCHT did not. The synergic effects of spironolactone and YCHT may lead to autoregulation in RAAS of angiotensin receptors on vasodilation and natriuresis while co-administrating.

Based on the pharmacokinetic and pharmacodynamic results of coadministration of 1 g/kg and 3 g/kg YCHT with spironolactone, the interactions of spironolactone and YCHT observed in decreasing urinary sodium–potassium and water excretion were attributed to the decreased metabolite of spironolactone as well as a possible synergistic contribution in ACE2 upregulation to subsequent sodium water homeostasis. Treatment of bile duct-ligated rats with 1 g/kg YCHT and spironolactone caused pharmacokinetic interactions in the elimination half-life and decreased urinary sodium and potassium excretion. Furthermore, treatment with 3 g/kg YCHT and spironolactone also decreased urinary sodium potassium excretion and further decreased urine output, as well as the metabolites of spironolactone with a maintained maximum excretion time. The coadministration of YCHT and spironolactone to bile duct-ligated rats produced diminished metabolites of spironolactone, and the urine sodium and urine output in our experiment could be used to investigate the RAAS mechanism of related hormones and enzymes based on the co-contribution of the antifibrosis pathway of YCHT and spironolactone for therapeutic purposes in the future.

## 5 Conclusion

The combined pharmacokinetic and pharmacodynamic results showed that solitary YCHT treatment, although it caused variation in the urinary sodium-to-potassium ratio at a dose of 1 g/kg, did not change the urine output in bile duct-ligated rats. The coadministration of 1 g/kg YCHT and spironolactone also resulted in a steady urinary sodium-to-potassium ratio compared to spironolactone alone and urine output without obvious alterations in pharmacokinetics. The interaction was obvious when 3 g/kg YCHT was administered, which caused diminished urine output and spironolactone metabolites. Based on the interactions between spironolactone and YCHT, the use of YCHT and spironolactone to treat hepatic diseases could avoid sodium–potassium imbalance, and this should be paired with adjusting the dosage of YCHT and monitoring its diuretic effects in clinical use.

## Data Availability

The raw data supporting the conclusion of this article will be made available by the authors, without undue reservation.

## Abbreviations

ACE2, angiotensin-converting enzyme-2; AT1R, angiotensin II type 1 receptor; AURC, the area under the urinary excretion rate curve; BDL, bile duct ligation; Cum, cumulative amount; HPLC-UV, high-performance liquid chromatography-ultraviolet; Ke, elimination rate constant; RAAS, renin–angiotensin–aldosterone system; R_max_, the maximum rate of excretion; SD rats, Sprague Dawley rats; T_max_, time to the maximum rate of excretion; UHPLC–MS/MS, ultra-performance liquid chromatography–tandem mass spectrometer; YCHT, Yin-Chen-Hao-Tang.
